# Histone Deacetylase 7‐Derived 7‐Amino Acid Peptide Increases Skin Wound Healing via Regulating Epidermal Fibroblast Proliferation and Migration

**DOI:** 10.1111/jcmm.70209

**Published:** 2024-11-27

**Authors:** Huina Liu, Hua Li, Xuefeng Bai, Yue Zhao, Yannan Cai, Huiqing Pan, Linyan Guo, Kun Liu, Qian Liu, Xiaochun Huang, Anna Zampetaki, Andriana Margariti, Lingfang Zeng, Ting Cai

**Affiliations:** ^1^ Ningbo No.2 Hospital Ningbo China; ^2^ Ningbo Institute of Life and Health Industry University of Chinese Academy of Sciences Ningbo China; ^3^ School of Cardiovascular and Metabolic Medicine and Sciences, Faculty of Life Science and Medicine King's College London London UK; ^4^ Ningbo Women and Children's Hospital Ningbo China; ^5^ Burdon Sanderson Cardiac Science Centre and BHF Centre of Research Excellence, Department of Physiology, Anatomy and Genetics University of Oxford Oxford UK; ^6^ Department of Geriatric Chengdu Fifth People's Hospital Chengdu China; ^7^ GDK Med Ltd. London UK; ^8^ School of Medicine, Dentistry and Biomedical Sciences The Wellcome‐Wolfson Institute of Experimental Medicine Belfast UK

**Keywords:** 7‐amino acid peptide, CDK6, CTNNB nuclear translocation, CTNND1 phosphorylation, PDGFRB, Skin wound healing

## Abstract

Due to the complexity of wound healing, how to achieve successful healing is a significant clinical challenge. In this study, we found that the histone deacetylase‐7‐derived 7‐amino acid peptide (7A, MHSPGAD), especially its phosphorylated version 7Ap (MH[pSer]PGAD), increased dermal fibroblast cell HDFα proliferation and migration via elevated delta‐catenin (CTNND1) serine phosphorylation‐mediated beta‐catenin (CTNNB) nuclear translocation and subsequent upregulation of c‐Myc and cyclin D1 expression. 7Ap physically interacted with platelet‐derived growth factor receptor (PDGFR) and increased PDGFR interaction with cyclin‐dependent kinase 6 (CDK6). The *PDGFR* siRNA or *CDK6* siRNA knockdown ablated 7AP‐induced CTNND1 phosphorylation and subsequent c‐Myc/cyclin D1 expression, indicating a novel 7Ap‐PDGFR‐CDK6‐CTNND1/CTNNB signal pathway in regulating fibroblast proliferation and migration. Furthermore, 7Ap increased human umbilic vein endothelial cell proliferation and tube formation, suggesting an angiogenic effect. In a full‐thickness excision wound rat model, the local administration of 50 ng/mL of 7Ap in hydrogel exerted a similar effect as 1 μg/mL vascular endothelial growth factor on accelerating wound healing, featured by enhanced fibroblast proliferation and migration, collagen deposition, and increased new vessel formation during the early phase of wound healing. Taken together, this study not only elicited a novel signal pathway in fibroblast proliferation but also paved an avenue to develop 7Ap as a treatment option for skin wound healing.

## 1 Introduction

The growing elderly and diabetic populations pose a significant challenge to global health and have emphasized the complexities of wound healing as a major global health issue [1, 2]. This multifaceted and dynamic restorative process unfolds across four distinct yet interconnected phases: homeostasis, inflammation, proliferation, and remodeling [3, 4]. Any disruption during this continuum, such as aberrant inflammation, diminished cell proliferation and migration, or compromised expression of growth factors, can lead to impaired wound healing and subsequent complications. Notably, fibroblasts, pivotal players in wound repair and regeneration, orchestrate these processes by generating a sophisticated extracellular matrix and establishing signalling niches through biophysical and biochemical cues [5, 6]. Ongoing research underscores the tight correlation between fibroblast dysfunction and the development of non‐healing wounds [7]. The success of wound healing critically hinges on the timely accumulation and activation of fibroblasts, orchestrators of granulation tissue deposition, and wound contraction [8]. Consequently, the modulation of fibroblast activity, especially during the proliferative phase, emerges as a crucial mechanism for clinicians to influence the trajectory of wound healing outcomes [9]. Central to these regulatory mechanisms is the Wnt/β‐catenin pathway, a principal signalling cascade intricately involved in wound healing. This pathway plays a pivotal role in governing cell proliferation, differentiation, neoangiogenesis, and tissue regeneration [10]. Noteworthy is the upregulation of Wnt/β‐catenin signalling, shown to augment fibroblast cell proliferation and migration, inducing their trans‐differentiation into myofibroblasts [10, 11]. As a result, the activation of the Wnt/β‐catenin pathway has garnered attention as a promising avenue for the development of wound healing agents in recent years [12, 13]. This paradigm shift in therapeutic exploration highlights the potential of targeting the Wnt/β‐catenin pathway to enhance fibroblast function and, consequently, advance the field of wound healing research.

Among the various therapeutic strategies for skin wound repair, growth factor‐based therapies, including platelet‐derived growth factor (PDGF), fibroblast growth factor (FGF), epidermal growth factor (EGF), and vascular endothelial growth factor (VEGF), have garnered widespread attention in clinical trials [14, 15, 16]. These growth factors exhibit high therapeutic efficacy in promoting wound healing. However, their practical application is often hampered by challenges such as limited absorption, low stability, poor penetration, and associated adverse effects, ranging from local allergic reactions to excessive cell growth that culminates in scar development [17]. In stark contrast, therapeutic peptides present a compelling alternative, circumventing these issues owing to their low molecular weight, which ensures low immunogenicity and relatively rapid metabolism [18]. Additionally, their competitive advantage lies in lower production costs, underscoring their potential as a more viable option for addressing the limitations of traditional growth factor therapies.

Histone Deacetylase 7 (HDAC7), a representative of class II HDACs, demonstrates expression in the vascular endothelium and plays a pivotal role in maintaining vascular integrity during early embryogenesis [19]. Building upon our earlier investigations, we have identified a 7‐amino acid peptide (7A, MHSPGAD) translationally derived from the 5′ untranslated region (UTR) of mouse *Hdac7* mRNA in response to VEGF or pathological stimuli. Intriguingly, the 7A peptide serves as a phosphate carrier, actively participating in signal transduction. Local administration of the synthetic 7A peptide, particularly its phosphorylated counterpart (7Ap, MH[pSer]PGAD), exerts a pronounced effect on stem cell antigen‐1 positive (Sca1+) vascular progenitor cell (VPC) migration and facilitates their differentiation towards an endothelial cell lineage. This, in turn, contributes significantly to vascular injury repair and promotes angiogenesis in ischemic tissues [20, 21].

Given the therapeutic potential of 7Ap in vascular repair and regeneration, we hypothesize that it may have a positive impact on the wound‐healing process. This investigation explores the effect of 7Ap on wound healing using a full‐thickness excision wound rat model. Furthermore, our study aims to clarify the specific molecular mechanisms through which 7Ap activates fibroblasts, providing insight into its targeted pathways in wound healing.

## 2 Materials and Methods

### 2.1 Materials

All cell culture media, supplements, and serum were purchased from ThermoFisher Scientific. Antibodies against p120‐catenin (sc‐23873), PDGFRβ1(sc‐19995), CCND1 (sc‐8396), c‐Myc (sc‐40), β‐catenin (sc‐7199) and GAPDH (sc‐25778) were purchased from Santa Cruz Biotech, antibodies against pSer (P3430), pThr (MABS499), pTyr (SAB5700563), α‐tubulin (T8203) and LaminB1 (ZMS1128) were from Sigma, antibodies against CTNND1 (12180‐1‐AP) and CDK6 (14052‐1‐AP) were from Proteintech, antibodies against Ki‐67 (ab279653), CD31 (ab182981) and c‐Myc (ab32072) were from Abcam, and antibody against delta 1 Catenin (Phospho‐Ser268) was from Biorbyt. All secondary antibodies were from Dako Cytomation (Agilent, Santa Clara, USA). Inhibitors PD98059 and SU5416 were purchased from Sigma and dissolved in DMSO. All other chemicals were also from Sigma.

### 2.2 Cell Culture

HDFα (human adult primary dermal fibroblast, PCS‐201‐012) cells were purchased from ATCC and maintained in Dulbecco's Modified Eagle's Medium supplemented with 10% fetal bovine serum and penicillin/streptomycin. The cells were split at a ratio of 1:4 every three days. HUVECs were purchased from ATCC and maintained in M199 medium supplemented with 10% FBS, 1 ng/mL β‐endothelial cell growth factor (E1388, Merck Life Science Ltd.), 3 μg/mL endothelial growth supplement from bovine neural tissues (E2759, Merck), 10 U/mL Heparin (H3149, Merck), 1.25 μg/mL thymidine (T1895, Merck), and 100 U/mL penicillin/streptomycin. HEK293 and HeLa were purchased from ATCC and maintained with the same medium as HDFα.

### 2.3 Cell Proliferation

Cell proliferation was performed with an MTT assay and cell counting. For the MTT assay, HDFα cells were seeded in 96‐well plates at 1 × 10^4^ cells/0.1 mL/well with 1 ng/mL peptides in triplicates. The medium was refreshed every other day. On day 3, the MTT assay was performed according to the protocol provided. For cell counting, HDFα cells were seeded in 12‐well plates at 5 × 10^4^ cells/1 mL/well with 1 ng/mL peptides in triplicates. The medium was refreshed every other day. At day 5, the cells were trypsinized to single cell suspension and subjected to cell counting using a multisizer 3 coulter counter (Beckman Coulter) according to the manufacturer's instructions. For both assays, 1% BSA was included as vehicle control. HUVEC, HEK293 and HeLa cell proliferation was assessed by cell population doubling (CPD) assays as described in figure legends. CPD = (log_10_F − log_10_I)/log_10_2, F: final cell number; I initial cell number.

### 2.4 Cell Migration Assays

Cell migration was assessed using transwell migration assays and a wound healing model. For transwell migration assay, 3 × 10^4^ HDFα cells in 200 μL serum‐free DMEM were seeded in the insert of a 24‐well transwell with an 8 μm pore and placed in the 24‐well transwell plate with the holder containing 600 μL/well of DMEM plus 1 ng/mL peptide. The whole set was placed in a cell culture incubator and incubated for 6 h, followed by staining with crystal violet. After the removal of the cells inside the insert, the migrated cells on the outside membrane of the insert were observed under the microscope. Images were taken, and the cell number was calculated using ImageJ. For the wound healing model, HDFα cells were seeded in 12‐well plates and cultured until fully confluent. The wound was introduced by scratching with a 1 mL tip to make a cross; marks were created with needle puncture punctures in all four directions. After the cell debris was removed, DMEM containing 0.5% FBS and 1 ng/mL peptide. The image was taken at 0 and 16 h from all four directions of each cross mark. Migrated cells/cm^2^ were calculated thereafter. The experiments for both assays were performed in triplicate.

### 2.5 Tube Formation

BD Matrigel Matrix Growth Factor Reduced (354230, BD Biosciences) was thawed on ice overnight. Eighty microlitre per well of Matrigel solution was added to 96‐well plates and solidified at 37°C for 30 min. HUVECs were detached by trypsin to make a single‐cell solution in M199 supplemented with 10% FBS and adjusted to a final concentration of 1.5 × 105 cells/mL. One hundred μL of cell suspension was added to each well containing Matrigel. 20μL of 100 ng/mL 7Sp or 7Ap or 500 ng/mL VEGF were added in triplicate. Images were taken at 5 or 24 h using 10× lenses; up to three views were taken for each well. The experiments were performed three times independently. Branches of the tubes were analysed. All views from 5 and 24 h for each well and all wells from different repeats for each type of treatment were pooled together.

### 2.6 Cellular Fractionation

HDFα cells were pre‐treated with DMEM containing 0.5% FBS overnight, then treated with 1 ng/mL 7Ap with 7Sp included as a control for 2 h. For inhibitor experiments, the cells were pre‐treated with inhibitors one hour before peptide treatment. The cells were washed twice with cold PBS, scratched off, and resuspended in 200 μL/1 × 10^6^ cells cold hypotension buffer (10 mM Hepes‐KOH, pH 7.2, 1.5 mM MgCl2, 10 mM KCl plus Protease inhibitors) and incubated on ice for 15 min. 12.5 μL of 10% NP‐40 were added and vortex vigorously for 10 s, followed by spinning down at highest speed for 10 s. The supernatant was recovered as cytosolic fractions. The pellet was washed twice with cold PBS and resuspended in 100 μL hypotension buffer containing 0.625% NP‐40, sonicated for several seconds, and incubated on ice for 30 min, followed by spinning at the highest speed for 5 min. The supernatant was recovered as nuclear extract fractions. Protein level was measured with Bio‐Rad reagents.

### 2.7 Immunoprecipitation and Immunoblotting

Immunoprecipitation and immunoblotting were performed according to standard procedures described elsewhere. One milligram of lysate was used for immunoprecipitation together with 2 μg antibody and 10 μL protein‐G beads (Sigma), while 25 μg was used for input or direct immunoblotting.

### 2.8 Indirect Immunofluorescence Staining

Immunofluorescence staining was performed according to the standard procedure described elsewhere. Briefly, cultured cells or rat tissue cryo‐sections were fixed with 4% paraformaldehyde at room temperature for 15 min, permeabilized with 0.1% Triton X‐100 at RT for 15 min, followed by blocking with diluted swine serum (1:20) for 1 h, incubation with diluted primary antibodies (1 μg/mL) at 4°C overnight, and with secondary antibodies at room temperature for 2 h. The nucleus was counterstained with DAPI. Images were taken using an SP5 confocal microscope (Leica, Germany), and processed by Adobe Photoshop. Magnification was indicated in figure legends as scale bars.

### 2.9 Reverse Transcriptase and Quantitative Polymerase Chain Reaction (RT‐qPCR)

Total cellular RNA was extracted using the Qiagen RNeasy Kit according to the protocol provided. One microgram RNA was transcribed into cDNA using the Improm‐II reverse transcription system (Promega). Twenty nanogram cDNA (relative to the RNA amount) was amplified by a real‐time PCR SYBR master mix (Applied Biosystems). Primers for qPCR were designed with the Primer Express Software (Applied Biosystems) and listed as following: *c‐MYC*: 5′>cga cag cag ctc gcc caa gtc ctg< 3 ′ versus 5′>gca gaa ggt gat cca gac tct gac< 3 ′; *CCND1*: 5′>ctg ccg tcc atg cgg aag atc gtc 3 ′ versus 5′>cac cag gag cag ctc cat ttg cag 3 ′ and internal control 18S RNA: 5′>ata cat gcc gac ggg cgc tg 3 ′ versus 5′>gga gag ggg ctg acc ggg tt 3 ′.

### 2.10 Chromatin Immunoprecipitation

Chromatin immunoprecipitation (ChIP) was performed according to standard procedures described elsewhere. Briefly, HDFα cells were treated with 7Ap peptide as described above, with 7Sp included as a control. The cells were then treated with 1% formaldehyde at 37°C for 15 min to crosslink DNA with bound proteins, followed by neutralization with 0.2 M glycine at room temperature for 15 min. The cells were washed with cold PBS, harvested by scratching off, and subjected to cellular fractionation as described above. The nuclear pellet was resuspended in 200 μL Sonication buffer (50 mM Tris–HCl, pH 8.0, 0.1% SDS, 10 mM EDTA, plus protein inhibitors), sonicated at 30% power output for 4 × 10 s, then centrifuged at 10000 rpm for 5 min. The supernatant was recovered and diluted with 1 mL IP buffer (20 mM Tris–HCl pH 7.2, 100 mM NaCl, 10% glycerol, 0.1% NP‐40, 1 mM EDTA, plus protein inhibitors), precleared with 50 μL of single strand salmon sperm DNA saturated Protein A magnetic beads by incubation at 4°C for 15 min, followed by centrifuge at 4°C at 10000 rpm for 5 min. The supernatant was recovered, and incubated with 2 μg specific antibody or normal IgG at 4°C for 2 h, then 25 μL single strand salmon sperm DNA saturated Protein A magnetic beads were added and incubated further for 2 h. The beads were washed 5 times with IP Washing Buffer (20 mM Tris–HCl pH 7.2, 100 mM NaCl, 0.2% NP‐40, 1 mM EDTA). The supernatant of the normal IgG group was kept as input control. The immunoprecipitates were eluted from the beads by three rounds of gentle vortexing for 10 min in 100 μL of Elution Buffer (50 mM NaHCO3, 1% SDS). The elute was pooled and mixed with 200 μL of Proteinase K buffer (50 mM Tris–HCl, PH7.5, 0.3% SDS, 10 mM EDTA, 0.5 M NaCl, 1 mg/mL Proteinase K), and incubated at 60°C for 8 ~ 16 h. For the input control, SDS was adjusted to 0.7% with NaCl to 0.2 M, EDTA to 5 mM, and Proteinase K to 400 μg/mL. The solution was extracted once with phenol/chloroform, and the supernatant was mixed with 5 μg tRNA, 3 vol ethanol, and stored at −20°C overnight. DNA was pelleted by centrifuge at 12000 rpm for 5 min, washed once with 70% ethanol, and then dissolved in 30 μL ddH2O. Two microliters of DNA was applied to PCR to amplify DNA fragments in the promoter region with primer sets listed. For the input, the DNA was dissolved in 100 μL ddH2O, and diluted 1:100 for PCR. Primer sets: c‐Myc promoter: 5′>gctctccacttgcccctttta 3 ′ & 5′>gttcccaatttctcagcc 3 ′; CCND1 promoter: 5′>gactacaggggagttttgttg 3 ′ & 5′>tcggctctcgcttctgctg 3 ′; GAPDH promoter: 5′>tactagcggttttacgggcg 3 ′ & 5′>tcgaacaggaggagcagagagcga 3 ′.

### 2.11 siRNA Transfection

siRNA transfection was performed using GenMute Reagent (SignaGen laboratories) with some modifications to the protocol provided. Briefly, HDFα cells were detached by trypsin to make a single‐cell suspension (5 × 10^5^ cells/mL) in serum‐free DMEM. Five pmol of PDGFRB siRNA (PDGFRBsi, AM16708‐143751, ThermoFisher Scientific) or CDK6 siRNA (CDK6si, AM16708‐103566, ThermoFischer Scientific) or control siRNA (ctlsi, sc‐37007, Santa Cruz Biotech) was diluted into 100 μL 1× working solution (kit provided), mixed with 15 μL GenMute reagent, and incubated at room temperature for 15 min. The mixture was then added to 1 mL cell suspension, and incubated on a rotator at 37°C for 5 h. The transfected cells were then seeded in T25 mL flasks in 5 mL of complete growth DMEM and cultured for 72 h with medium refreshed every other day. After 72 h, the cells were pre‐treated with serum‐free medium overnight, then treated with 1 ng/mL 7Ap (with 7Sp included as control) for 2 h, followed by IP and/or WB as indicated in figure legends.

### 2.12 Animal Wound Healing Experiment

All animal studies were approved by the Institutional Animal Ethics Committee Ref No. GK‐2023‐LW‐1002 and were conducted by relevant guidelines and regulations. 27 Male Sprague Dawley (SD) rats (aged 8–12 weeks) were purchased from Hangzhou Moldhar Biotechnology Co. Ltd. and maintained for 1 week prior to initializing the experiments. A full‐thickness excision wound (dimension 1.5 × 1.5 cm) was punched onto the dorsal skin using a biopsy punch as described previously [22]. Rats were randomized into three treatment groups: Control (PBS), Positive control (1 μg/mL VEGF), and 50 ng/mL 7Ap group. The drugs were loaded in 25% Pluronic F‐127 gel (Sigma, MO, USA) and were delivered one time every three days for 14 days of therapy. The wound was photographed and measured by ImageJ software (National Institutes of Health, MD, USA). Wound closure was calculated using the following equation: wound area (%) = At /A0 × 100, where A0 is the initial wound size, and At is the wound size at the indicated times. Rats were sacrificed at postoperative days 3, 7, and 14 were harvested from the outer edge of the entire wound for histological analysis.

### 2.13 Statistical Analysis

Data are expressed as boxes (first and third quartiles) and whiskers (maximum and minimum). The middle line in the box represents the median. Mean ± SD was used. All data are analysed using GraphPad Prism 7 software with *t*‐test for pairwise comparisons or analysis of variance, and significance was depicted by asterisks, *p < 0.05, **p < 0.01.

## 3 Results

### 3.1 Local Delivery of 7Ap Peptide Accelerates Wound Healing In Vivo

**FIGURE 1:**
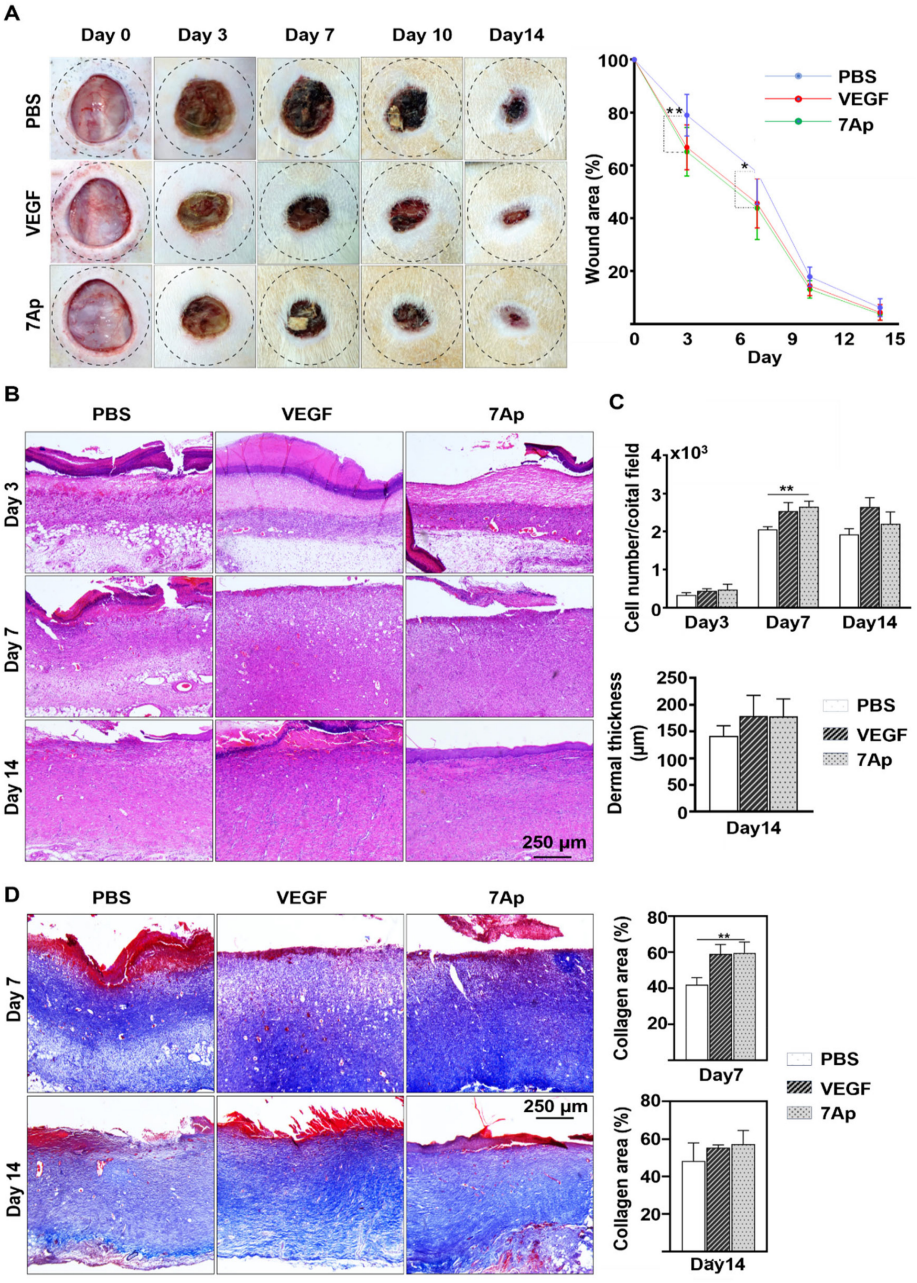
7Ap Peptide accelerated wound healing in vivo. (A) Wound area in each group was imaged and measured at 0 (*n* = 9), 3 (*n* = 9), 7 (*n* = 6), 10 (*n* = 3), and 14 (*n* = 3) days after surgery and presented as the wound area %. Left: representative images, right: wound area %. (B) Representative images of dermal layers of the wound tissues stained with H&E at day 3, 7, and 14 post‐surgery (*n* = 3). (C) Statistic analysis of dermal cell numbers (upper) and dermal thickness (lower) (*n* = 3). (D) Representative images of Masson's trichrome at days 7 and 14 post‐surgery (left) and collagen deposition analysis (right) (*n* = 3). *p < 0.05; **p < 0.01.

**FIGURE 2:**
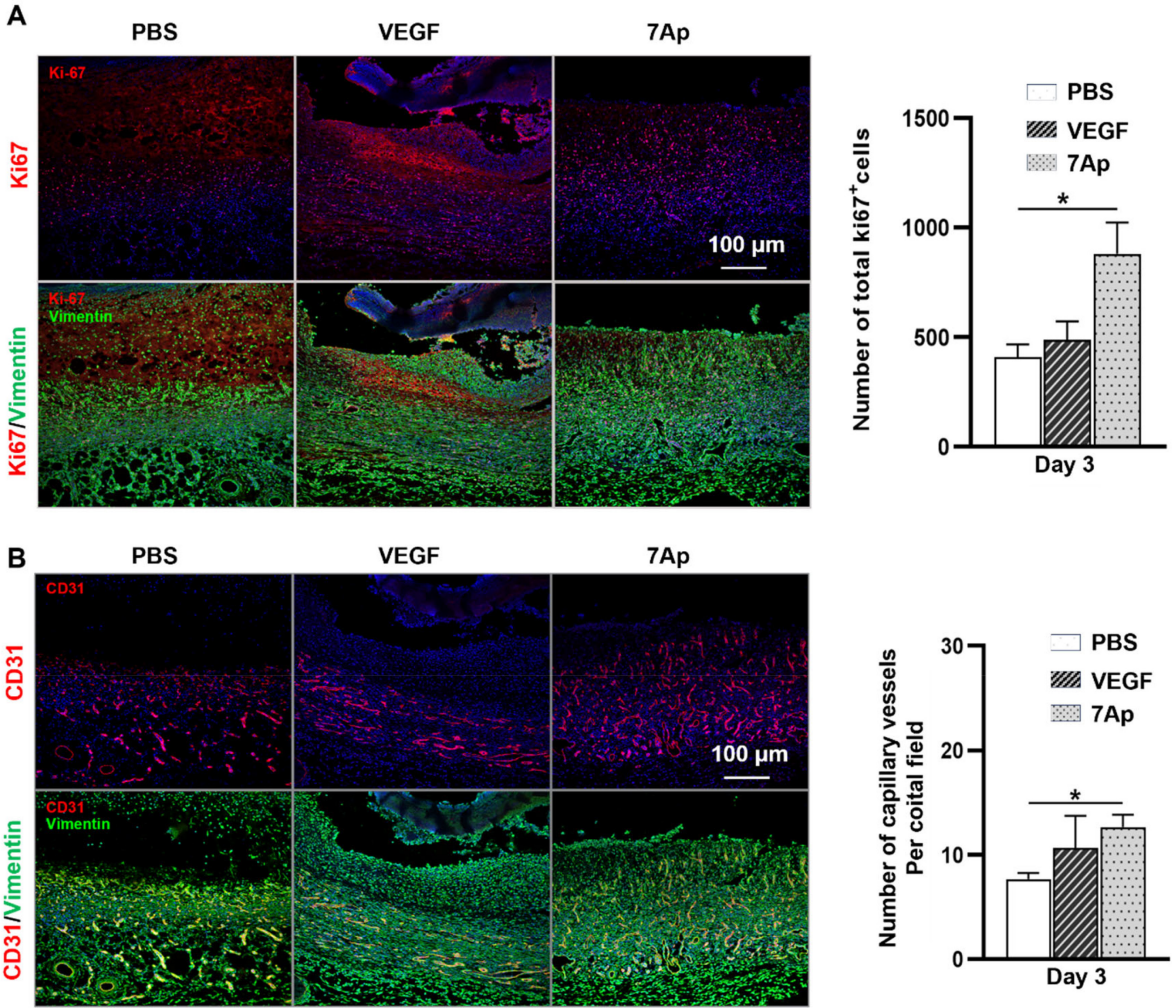
7Ap increased cell proliferation and angiogenesis in vivo. Immunofluorescence staining with anti‐Ki67 (A) and CD31 (B) was performed on sections in the dermal layers of the wound tissues at day 3 post‐surgery for cell proliferation (A) and angiogenesis (B). Proliferating cells and capillary vessels were analysed and presented in the right of the panels respectively. *n* = 3. *p < 0.05.

To evaluate the in vivo efficacy of 7Ap on wound healing, a full‐thickness excision wound was created on the dorsal region of a rat (Figure S1). A locally applied Pluronic F‐127 gel containing 7Ap peptide (50 ng/mL) served as the experimental treatment, with PBS and VEGF (1 μg/mL) serving as the vehicle and positive controls, respectively. As depicted in Figure 1A, 7Ap demonstrated a comparable acceleration of wound healing to VEGF, as evidenced by a significant reduction in wound area compared to the PBS control. By days 3 and 7, the 7Ap treatment group exhibited a significantly diminished wound area compared to the control group. The pivotal role of fibroblast proliferation and migration in skin tissue regeneration is well‐established [3, 4]. Haematoxylin & Eosin (H&E) and Masson's trichrome staining revealed that 7Ap‐treated wounds displayed increased cell numbers, a thicker dermis and augmented collagen deposition in the dermal layers compared to control groups (Figure 1B–D). Immunofluorescence staining further confirmed higher expression of the proliferation marker ki67 in the 7Ap treatment group (Figure 2A). Angiogenesis, a critical indicator of successful wound healing, was assessed through immunofluorescent detection of CD31 expression in the dermal layers at day 3 post‐surgery. As expected, both 7Ap and VEGF treatment groups showed a higher number of capillary vessels in the dermal layers compared to the PBS control (Figure 2B). In summary, 7Ap enhances wound healing by promoting angiogenesis, fibroblast proliferation, and migration. Notably, considering its low dosage (50 ng/mL vs.1 μg/mL VEGF) and comparable effectiveness to VEGF, 7Ap shows significant potential as a therapeutic agent for wound healing.

### 3.2 7Ap Increases HDFα Cell Proliferation and Migration via β‐Catenin Nuclear Translocation

**FIGURE 3:**
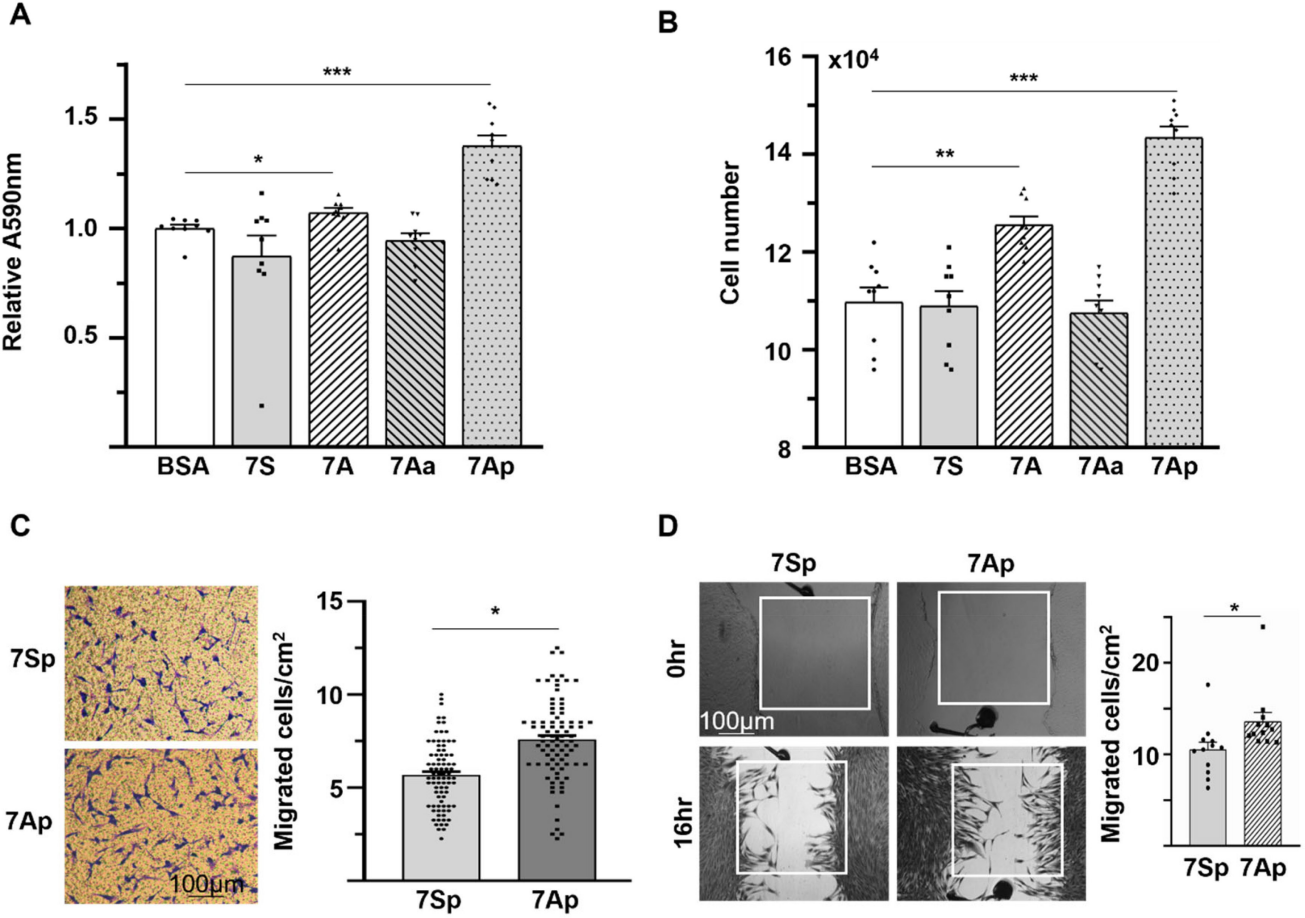
7Ap increased HDFα cell proliferation and migration. (A, B) 1 × 10^4^ cells/0.1 mL/well were seeded in 96‐well (A) or 5 × 10^4^ cells/1 mL/well in 12‐well (B) plates with 10 ng/mL peptides in triplicate with medium refreshment every other day, followed by MTT assay at day 3 (A) or cell counting at day 5 (B). 1% BSA was included as vehicle control. Data presented were three independent experiments (*n* = 9). (C) transwell migration assay was performed with 3 × 10^4^ cells/insert with 10 ng/mL peptide in holder in triplicate in DMEM medium containing 0.5% FBS for 8 h. Three images were taken from each insert with 4× lenses, and cells were counted from random three 2 × 2 cm areas of each image. A total of 81 areas were calculated from three independent experiments and plotted in the right (*n* = 81). Representative images were presented on the left. (D) wound healing was performed in four wells of confluent HDFα with 10 ng/mL peptides in DMEM medium containing 0.5% FBS for 16 h. Images were taken at 0 and 16 h. Data presented were representative or mean of three independent experiments (*n* = 12). 7S; HPHASGD, scrabbled control; 7A: MHSPGAD; 7Aa: MHAPGAD; 7Sp: HPHA(pS)GD; 7AP: MH(pS)PGAD. *p < 0.05; **p < 0.01; ***p < 0.001.

As delineated above, the increased fibroblast density observed in the 7Ap‐treated group suggests a potential enhancement of fibroblast migration and proliferation within the injured area. To explore this hypothesis, in vitro proliferation and migration assays were conducted on human primary adult dermal fibroblast HDFα cells. The 7A peptide (MHSPGAD), particularly its phosphorylated iteration 7Ap (MH[pS]PGAD), significantly increased HDFα cell proliferation, as demonstrated by MTT assay and cell counting analysis (Figure 3A,B). This effect was abolished when the serine residue was substituted with alanine (7Aa, MHAPGAD), highlighting the role of serine phosphorylation. Subsequent experiments were focused on 7Ap, and both Transwell and wound healing model assays confirmed the enhancement of the augmentation of HDFα cell migration (Figure 3C,D). These findings strongly support that fibroblast proliferation and migration are crucial to the 7Ap‐accelerated healing of skin wounds.

**FIGURE 4:**
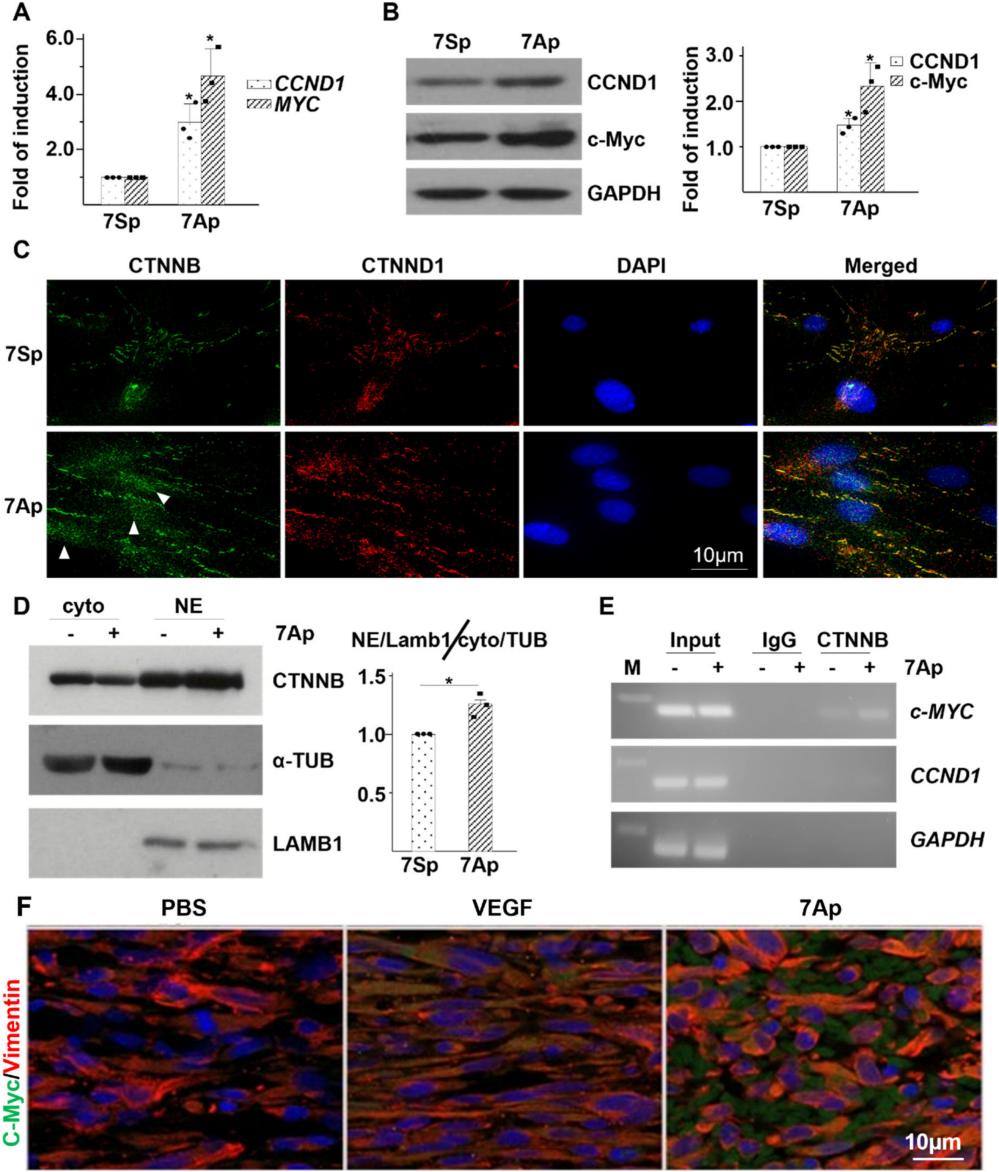
7Ap regulated cell proliferation via Wnt signal pathway in vitro and in vivo. HDFα cells were treated with 10 ng/mL 7Ap in serum free medium for 2 h or 6 h, followed by qRT‐PCR (A, 6 h, Fold of induction was defined as ratio of target gene in 7Ap group to 7Sp group with that of 7Sp group set as 1.0.), WB (B, 6 h Fold of induction was defined as ratio of target protein/GAPDH in 7Ap group to 7Sp group with that of 7Sp group set as 1.0.), immunofluorescence staining with antibodies indicated and DAPI included to counterstain nuclei (C, 2 h, arrowheads indicated nuclear CTNNB), cellular fractionation (D, 2 h, NE/Lamb1/cyto/TUB was the ratio of nuclear (normalized to laminB1) to cytosolic (normalized to α‐tubulin) CTNNB with that of 7Sp group set as 1.0.) and chromatin‐immunoprecipitation plus PCR with primer sets of target genes indicated (E, 6 h). (F) Representative images of Immunofluorescence staining for c‐Myc (green) andvimentin (red) in the dermal layers of the wound tissues at day 3 post‐surgery. The data presented were representative images or mean of three independent experiments. *n* = 3. *p < 0.05.

Given the established correlation of Cyclin D1 and c‐Myc with cell proliferation [23], the impact of 7Ap on HDFα cells was assessed. The results showed that 7Ap increased Cyclin D1 and c‐Myc expression at both mRNA and protein levels in HDFα cells, as demonstrated by a quantitative reverse transcriptase polymerase chain reaction (qRT‐PCR) assay and Western blot analysis (Figure 4A,B). Considering the regulation of Cyclin D1 and c‐Myc by the Wnt signalling transcription factor β‐catenin (CTNNB) [23, 24], further investigations were conducted. 7Ap induced CTNNB nuclear translocation, as indicated by immunofluorescence staining and cellular fractionation (Figure 4C,D). Further chromatin immunoprecipitation (ChIP) assay revealed increased CTNNB binding to the c‐Myc and CCND1 promoters by 7Ap (Figure 4E). In wound dermal fibroblasts, 7Ap and VEGF treatments similarly upregulated c‐Myc compared to the vehicle control (Figure 4F). Collectively, these results suggest that 7Ap triggers CTNNB nuclear translocation and gene expression in a Wnt ligand‐independent manner, thereby promoting fibroblast cell proliferation both in vitro and in vivo.

### 3.3 CTNND1 Phosphorylation Is Required for 7Ap‐Induced CTNNB Nuclear Translocation

**FIGURE 5:**
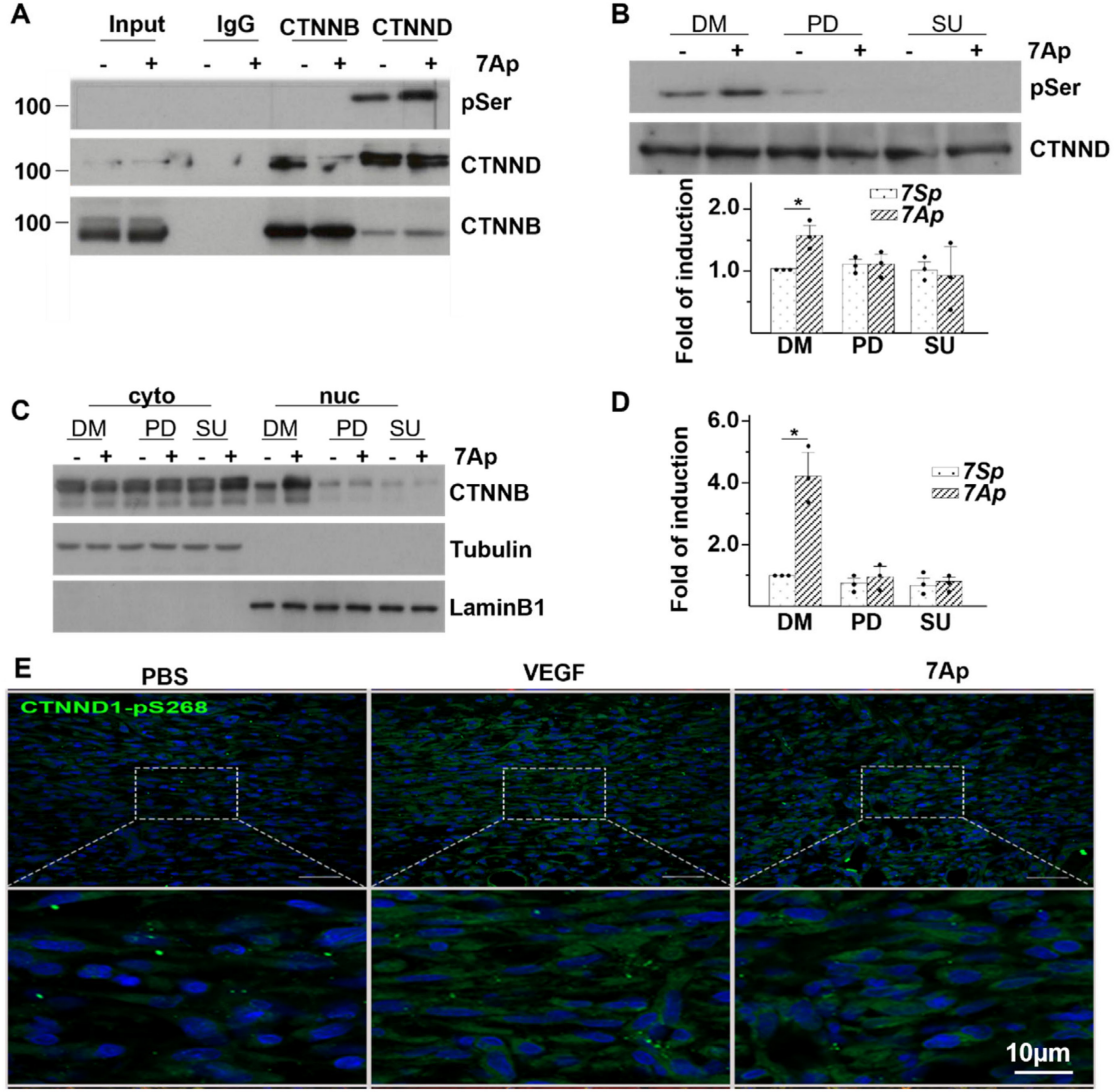
CTNND1 serine phosphorylation is essential for CTNNB nuclear translocation. (A) HDFα cells were treated with 10 ng/mL 7Ap in serum free medium for 2 h, followed by IP plus WB with antibodies indicated. 7Sp was included as control. (B‐D). HDFα cells were pre‐treated with 10 μM PD98059 (PD) or 5 μM SU5416 (SU) in serum free medium for 1 h, then treated with 1 ng/mL 7Ap in serum free medium in the presence of inhibitors for 2 h, followed by IP with anti‐CTNND1 plus WB with antibodies indicated to show CTNND phosphorylation (B), cellular fractionation plus WB with antibodies indicated to show CTNNB nuclear translocation (C) and qRT‐PCR analysis of CCND1 gene expression (D) DMSO (DM) was included as vehicle control. (E) Representative images of Immunofluorescence staining for phosphorylated CTNND1 in the dermal layers of the wound tissues at day 3 post‐wounding. Data presented were representative images or mean of three independent experiments. *p < 0.05.

The adherens junction (AJ) complex mediates adjacent cell communication and controls the contact inhibition of cell proliferation and migration [25, 26, 27]. Catenins are key components of the AJ complex, including alpha, beta, gamma, and delta (CTNND1) catenins [25, 27, 28, 29]. The stability of the AJ complex is known to be regulated by the phosphorylation of catenin proteins [30, 31]. In this study, we investigated the phosphorylation status of CTNNB and CTNND1 following 7Ap treatment using an immunoprecipitation (IP) assay. As illustrated in Figure 5A, our findings indicate that 7Ap induced serine phosphorylation in CTNND1 but not in CTNNB, while threonine and tyrosine phosphorylation were not observed. This treatment also reduced the interaction between CTNNB and CTNND1. Our previous research has shown that 7Ap can transfer its phosphate group to target proteins like 14‐3‐3γ [20]. Additionally, it has been reported that upstream receptor tyrosine kinases (RTKs) and mitogen‐activated kinases (MAPKs) are involved in CTNND1 phosphorylation [32, 33, 34]. To elucidate the mechanism by which 7Ap mediates CTNND1 phosphorylation, we employed the RTK inhibitor SU5416 [35] and the MAPK inhibitor PD98059 [36]. As shown in Figure 5B, both inhibitors abolished 7Ap‐induced CTNND1 phosphorylation, suggesting that this process requires upstream RTK and MAPK. Further experiments revealed that both inhibitors attenuated 7Ap‐mediated CTNNB nuclear translocation and CCND1 expression at the mRNA level (Figure 5C,D), indicating that CTNND1 phosphorylation is essential for CTNNB nuclear translocation and downstream gene transcription. Moreover, the upregulation of CTNND1 phosphorylation by 7Ap in dermal fibroblasts in wounds was detected by anti‐phospho‐CTNND1S268 antibody (Figure 5E). In summary, CTNND1 phosphorylation appears to play a significant role in 7Ap‐regulated fibroblast migration and proliferation in vitro and in vivo.

### 3.4 PDGFRB1‐CDK6 Pathway Is Involved in 7Ap‐Mediated CTNND1 Phosphorylation

**FIGURE 6:**
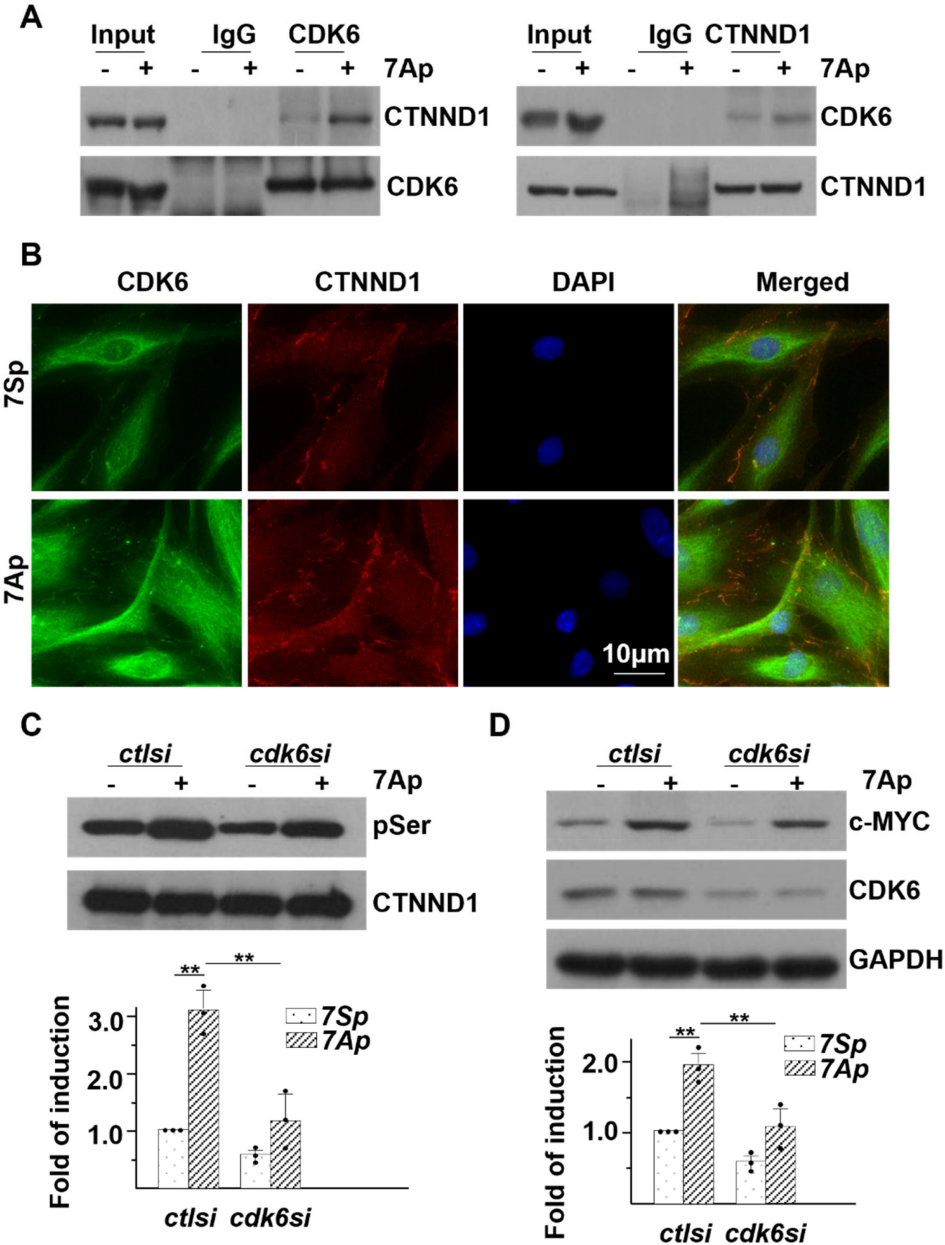
CDK6 is an upstream kinase for 7Ap‐mediated CTNND1 serine phosphorylation. (A, B) HDFα cells were treated with 10 ng/mL 7Ap in serum free medium for 2 h, followed by IP plus WB (A) or IF (B, DAPI included for nucleus counterstaining) with antibodies indicated. (C, D) HDFα cells were transfected with control siRNA (*ctlsi*) or *CDK6* siRNA (*cdk6si*) for 72 h, pre‐treated with serum free medium for 24 h and then treated with 10 ng/mL 7Ap for 2 h (C) or 6 h (D), followed by IP with anti‐CTNND1 plus WB (C) or WB (D) with antibodies indicated. 7Sp was included as control. Data presented were representative images or mean of three independent experiments. **p < 0.01.

Numerous phosphorylation sites have been documented in CTNND1 proteins, including serine (Ser), threonine (Thr), and tyrosine (Tyr) residues [31, 37, 38]. As elucidated earlier, 7Ap specifically exhibited an elevation in Ser phosphorylation. Therefore, our investigation focused on the serine phosphorylation of CTNND1. In vivo studies in rats demonstrated that CTNND1‐pS268 was upregulated by 7Ap, however, a comparable upregulation was not observed in HDFα cells, potentially attributable to environmental differences. To identify the phosphorylation sites and explore potential upstream RTK‐MAPK pathways, we conducted immunoprecipitation (IP) with anti‐CTNND1 coupled with proteomics analysis in 7Ap‐treated HDFα cells. Unexpectedly, none of the known serine phosphorylation sites were detected. Instead, we discovered four novel serine phosphorylation sites within three peptide fragments: SMGYDDLDYGMMS ^300^DYGTAR, ALS ^713^AIADLLTNEHERVVKAASGAL and EEIQMS ^891^NM GS ^895^NTKSLDNN YSTPNER (phosphorylation sites are underlined). Further investigation is required to characterize the exact serine residues involved. Several serine–threonine kinases were identified from the pool of CTNND1‐associated proteins. Notably, the association between cyclin‐dependent kinase 6 (CDK6) and CTNND1 was significantly enhanced by 7Ap treatment, as confirmed by co‐immunoprecipitation (co‐IP) and immunofluorescence (IF) assays (Figure 6A,B). siRNA‐mediated CDK6 knockdown significantly attenuated, though not entirely abolished, 7Ap‐induced CTNND1 phosphorylation and c‐Myc expression (Figure 6C,D), implying that CDK6 acts as an upstream kinase for CTNND1 phosphorylation and other kinases potentially involved.

**FIGURE 7:**
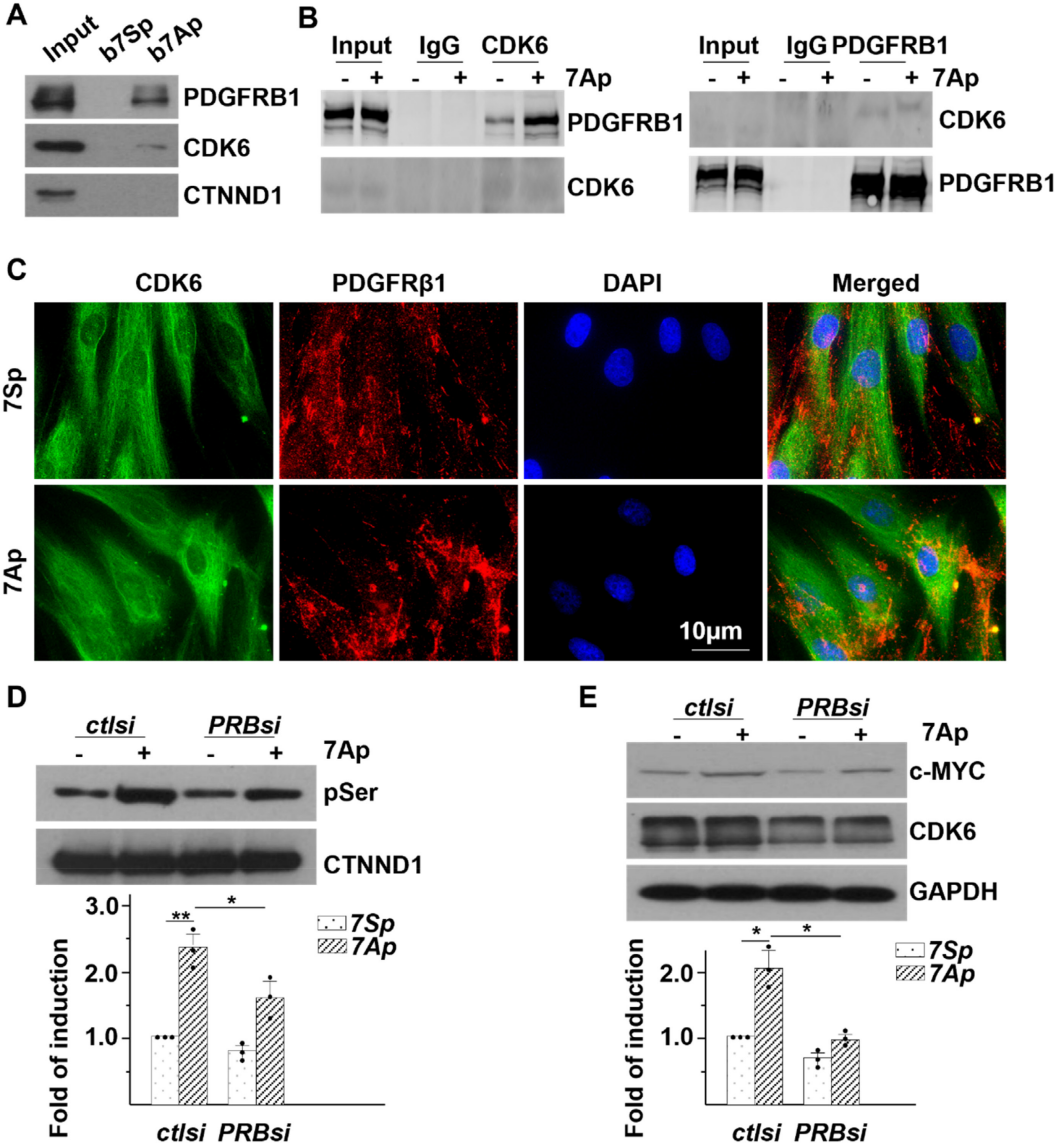
PDGFRB is an upstream RTK for 7Ap‐mediated CTNND1 serine phosphorylation. (A) HDFα cell lysate was incubated with biotin‐labelled 7Ap (b7Ap), followed by streptavidin magnetic beads pulldown and WB with antibodies indicated. b7Sp: biotin‐labelled 7Sp. (B, C) HDFα cells were treated with 10 ng/mL 7Ap in serum free medium for 2 h, followed by IP plus WB (B) or IF (C, DAPI included for nucleus counterstaining) with antibodies indicated. (D, E) HDFα cells were transfected with control siRNA (*ctlsi*) or *PDGFRB* siRNA (*PRB6si*) for 72 h, pre‐treated with serum free medium for 24 h and then treated with 1 ng/mL 7Ap for 2 h (D) or 6 h (E), followed by IP with anti‐CTNND1 plus WB (D) or WB (E) with antibodies indicated. 7Sp was included as control. Data presented were representative images or mean of three independent experiments. *p < 0.05; **p < 0.01.

Drawing from prior studies involving biotin‐labelled 7Ap‐mediated protein pulldown plus proteomics analysis in vascular progenitor cells [20], numerous receptor tyrosine kinases (RTKs) were identified, including the PDGF receptor beta (PDGFRB). Although PDGFRB has been reported to participate in CTNND1 phosphorylation [32], the specific serine residue identified in earlier research differed from our findings. Our current investigation confirmed the association of 7Ap with PDGFRB through a biotin‐labelled 7Ap‐mediated protein pulldown assay coupled with Western blotting (WB) (Figure 7A). Interestingly, we also detected an association between 7Ap and CDK6, but not with CTNND1 (Figure 7A). As anticipated, 7Ap increased the association between PDGFRB and CDK6, as shown by co‐IP plus WB and IF (Figure 7B,C). Furthermore, siRNA knockdown of PDGFRB significantly attenuated, though did not completely eliminate 7Ap‐induced CTNND1 phosphorylation and c‐Myc expression (Figure 7D,E). This suggests the involvement of PDGFRB in 7Ap‐mediated CTNND1 phosphorylation, possibly through other RTKs or direct interaction with MAPK(s) by 7Ap. Overall, the PDGFRB‐CDK6 pathway emerges as one of the contributory mechanisms in 7Ap‐mediated CTNND1 phosphorylation, CTNNB nuclear translocation, and the expression of genes associated with cell proliferation.

## 4 Discussion

Advancements in technology and scientific research have significantly enhanced our understanding of the complex wound‐healing process. Traditional materials and simplistic therapeutic strategies are increasingly viewed as inadequate, particularly in complex cases such as chronic wounds or extensive burns. Consequently, researchers are focusing on developing more effective therapeutics to improve healing outcomes. For instance, materials are being refined to better support tissue repair [39, 40], and advancements in drug delivery systems enable targeted drug release at the wound site to improve therapeutic outcomes and minimize side effects [41]. In this study, 7Ap was delivered by routine F‐127 pluronic gel. Further investigation on the combination of 7Ap with other carriers or delivery systems is required to improve healing outcomes for clinical implications. Angiogenesis has been identified as a promising therapeutic strategy for enhancing wound healing [42]. Previous studies have demonstrated that the exogenous synthetic 7Ap peptide contributes to the repair and regeneration of vascular injuries in ischemic tissues [20, 21]. A capillary vessel density increase was observed in 7Ap‐treated groups at the early stage of skin wound healing, suggesting an angiogenic effect of 7Ap. Indeed, the in vitro studies with the human umbilical vein endothelial cells (HUVECs) revealed that 7Ap increased HUVEC proliferation while without effect on other cell lines such as HEK293 and HeLa cess and that 7Ap significantly increased HUVEC tube formation ability (Figure S2A,B), which confirmed the angiogenic effect observed in vivo. Notably, the accelerated growth of new blood vessels at this initial phase transitions into the regression of excessive blood vessels at the later stages of angiogenesis during the wound healing process [43]. Excessive blood vessel growth has been associated with adverse outcomes such as scarring and the promotion of pathological processes including fibrosis, tumour progression, and various ocular diseases [43, 44]. The transient upregulation of angiogenesis induced by 7Ap within a limited timeframe ensures the delivery of essential nutrients and components for dermal fibroblast proliferation and migration, thereby facilitating wound healing.

In our study, the 7Ap peptide significantly promotes fibroblast cell proliferation and migration both in vivo and in vitro, which are crucial for effective wound healing. We found that 7Ap increased CTNND1 serine phosphorylation, facilitating CTNNB1 nuclear translocation and target gene transcription, thereby boosting fibroblast proliferation. CTNND1 is a key component of the AJ complex, binding to the juxtamembrane domain of VE‐cadherin to maintain AJ integrity [45]. Its role in regulating proliferation, differentiation, and barrier function in epidermal cells is well‐established [46]. Our findings indicate that 7Ap disrupts the AJ complex by phosphorylating CTNND1 in a manner independent of Wnt ligands, a process crucial for the release and nuclear translocation of CTNNB1, revealing a novel mechanism for activating the WNT signalling pathway. Mice deficient in CTNND1 exhibited a skin inflammatory disease, and impaired umbilical cord wound healing has been reported [46]. These results show that CTNND1 plays a crucial role in controlling normal epidermal/epithelial cell functions. CTNND1 is known to be phosphorylated by upstream receptor tyrosine kinases (RTKs) and mitogen‐activated protein kinases (MAPKs) [31, 37, 38], with numerous phosphorylation sites on serine, threonine, and tyrosine residues contributing to its diverse functions. In our study, serine residue phosphorylation was detected in 7Ap‐treated HDFα cells, revealing four previously unreported serine residues (S300, S713, S891, and S895), suggesting that 7Ap may trigger a unique pattern of CTNND1 phosphorylation. Interestingly, S268 phosphorylation in CTNND1 has been implicated in mesenchymal–epithelial transition in cancer cells via protein kinase C [47, 48]. Although the upregulation of S268 phosphorylation in CTNND1 was observed in 7Ap‐ and VEGF‐treated wounds in a rat model, but not detected in cultured HDFα cells. This discrepancy may arise from environmental variations. In vivo, 7Ap or VEGF might activate other cell types, releasing factors that regulate CTNND1 phosphorylation in dermal fibroblasts.

Our study underscores the PDGFRB‐CDK6 pathway as an influential route. In contrast to our previous findings on 7Ap‐mediated 14‐3‐3γ phosphorylation through direct interaction [20], we observed 7Ap enhances CTNND1 phosphorylation without directly interacting with CTNND1. Instead, 7Ap facilitates the interaction between CTNND1 and its upstream kinases, such as CDK6. The physical interaction of 7Ap with PDGFRB and CDK6, coupled with its demonstrated cellular entry, including potential nuclear access via endocytosis [20], suggests several plausible mechanisms within the 7Ap‐PDGFRB‐CDK6‐CTNND1 phosphorylation pathway. One possibility is that 7Ap acts as a ligand or phosphate donor, activating PDGFRB, which in turn triggers CDK6 activation and subsequent downstream cascade events. Alternatively, 7Ap may directly activate CDK6 as a ligand or donor. Regardless of the precise mode, the ultimate consequence is CTNND1 phosphorylation.

In summary, our study demonstrates that the topical administration of the 7Ap peptide effectively promotes cutaneous wound healing and improves healing quality through induction of new vessel formation in the early phase, stimulating fibroblast proliferation and migration and increasing collagen deposition. Mechanistically, 7Ap functions as a ligand or phosphate donor, activating the PDGFRB‐CDK6 pathway, which leads to the phosphorylation of CTNND1 at serine residues. This phosphorylation disrupts the adherens junction (AJ) complex and releases CTNNB. Subsequently, CTNNB translocates into the nucleus and directs the transcription of downstream target genes, such as c‐Myc and CCND1, thereby facilitating dermal fibroblast cell proliferation. Importantly, the 7Ap peptide exhibits a favourable safety profile, as no stimulation of cell proliferation was observed in HEK293 and HeLa cell lines during in vitro testing. Taken together, our findings suggest that the 7Ap peptide holds significant potential as a therapeutic alternative for skin wound healing.

## Author Contributions

**Huina Liu:** formal analysis (equal), funding acquisition (equal), supervision (equal), visualization (equal), writing – original draft (equal). **Hua Li:** formal analysis (equal), supervision (equal), visualization (equal), writing – original draft (equal). **Xuefeng Bai:** formal analysis (equal), supervision (equal), visualization (equal), writing – original draft (equal). **Yue Zhao:** supervision (supporting), visualization (supporting). **Yannan Cai:** supervision (supporting), visualization (supporting). **Huiqing Pan:** supervision (supporting), visualization (supporting). **Linyan Guo:** supervision (supporting), visualization (supporting). **Kun Liu:** writing – original draft (supporting). **Qian Liu:** writing – original draft (supporting). **Xiaochun Huang:** resources (equal). **Anna Zampetaki:** supervision (supporting), validation (supporting). **Andriana Margariti:** writing – original draft (supporting). **Lingfang Zeng:** conceptualization (equal), project administration (lead), writing – original draft (equal), writing – review and editing (equal). **Ting Cai:** conceptualization (equal), funding acquisition (equal), writing – review and editing (equal).

## Conflicts of Interest

The authors declare no conflicts of interest.

## Supporting information


**FIGURE S1.** An illustration of the procedure of the rat wound healing model. Full‐thickness excision wounds (diameter = 1.5 cm) were created on the back of SD rat. Each group was topically applied with 150 μL 25% Pluronic F‐127 gel containing either PBS, 1 μg/mL VEGF, or 50 ng/mL 7Ap peptide. *n* = 9.
**FIGURE S2**. 7Ap increased HUVEC proliferation and tube formation. (A) HUVECs (5 × 10^4^/well), HEK293 (5 × 10^4^/well), and HeLa (2 × 10^4^/well) were seeded in 6‐well plates at number indicate (initial) in complete growth factor and 10 ng/mL 7Sp or 7Ap for 72 h, followed by cell number counting (final). CPD (cell population doubling) was defined as (Log_10_F − Log_10_I)/Log_10_2, F and I indicated the final and initial number, respectively. *n* = 6. (B) HUVECs (1.5 × 10^4^cells/100 μL/well) in M199 medium supplemented with 10% FBS were seeded in 96 well plates containing 80 μL/well growth factor reduced Matrigel with 10 ng/mL 7Sp or 7Ap or 50 ng/mL VEGF, followed by imaging at 5 and 24 h. Left: representative images. Right: branch numbers analysis from views indicated. ns: no significant. ***:*p* < 0.001.

## Data Availability

The data that support the findings of this study are available from the corresponding author upon reasonable request.

## References

[1] C. K. Sen , “Human Wound and Its Burden: Updated 2020 Compendium of Estimates,” Advances in Wound Care 10 (2021): 281–292. 33733885 10.1089/wound.2021.0026 PMC8024242

[2] J. Holl , C. Kowalewski , Z. Zimek , et al., “Chronic Diabetic Wounds and Their Treatment with Skin Substitutes,” Cells 10 (2021): 655. 33804192 10.3390/cells10030655 PMC8001234

[3] J. Li , J. Chen , and R. Kirsner , “Pathophysiology of Acute Wound Healing,” Clinics in Dermatology 25 (2007): 9–18. 17276196 10.1016/j.clindermatol.2006.09.007

[4] G. C. Gurtner , S. Werner , Y. Barrandon , and M. T. Longaker , “Wound Repair and Regeneration,” Nature 453 (2008): 314–321. 18480812 10.1038/nature07039

[5] H. E. Talbott , S. Mascharak , M. Griffin , D. C. Wan , and M. T. Longaker , “Wound Healing, Fibroblast Heterogeneity, and Fibrosis,” Cell Stem Cell 29 (2022): 1161–1180. 35931028 10.1016/j.stem.2022.07.006 PMC9357250

[6] M. V. Plikus , X. Wang , S. Sinha , et al., “Fibroblasts: Origins, Definitions, and Functions in Health and Disease,” Cell 184 (2021): 3852–3872. 34297930 10.1016/j.cell.2021.06.024 PMC8566693

[7] A. Stunova and L. Vistejnova , “Dermal Fibroblasts—A Heterogeneous Population With Regulatory Function in Wound Healing,” Cytokine & Growth Factor Reviews 39 (2018): 137–150. 29395658 10.1016/j.cytogfr.2018.01.003

[8] J. Roman , “Fibroblasts‐Warriors at the Intersection of Wound Healing and Disrepair,” Biomolecules 13 (2023): 945. 37371525 10.3390/biom13060945 PMC10296409

[9] M. Zada , U. Pattamatta , and A. White , “Modulation of Fibroblasts in Conjunctival Wound Healing,” Ophthalmology 125 (2018): 179–192. 29079272 10.1016/j.ophtha.2017.08.028

[10] O. Burgy and M. Königshoff , “The WNT Signaling Pathways in Wound Healing and Fibrosis,” Matrix Biology: Journal of the International Society for Matrix Biology 68‐69 (2018): 67–80. 10.1016/j.matbio.2018.03.017 29572156

[11] A. P. Lam , A. S. Flozak , S. Russell , et al., “Nuclear Β‐Catenin Is Increased in Systemic Sclerosis Pulmonary Fibrosis and Promotes Lung Fibroblast Migration and Proliferation,” American Journal of Respiratory Cell and Molecular Biology 45 (2011): 915–922. 21454805 10.1165/rcmb.2010-0113OC PMC3262680

[12] P. Huang , R. Yan , X. Zhang , L. Wang , X. Ke , and Y. Qu , “Activating Wnt/β‐Catenin Signaling Pathway for Disease Therapy: Challenges and Opportunities,” Pharmacology & Therapeutics 196 (2019): 79–90. 30468742 10.1016/j.pharmthera.2018.11.008

[13] S. Choi , M. Yoon , and K. Y. Choi , “Approaches for Regenerative Healing of Cutaneous Wound with an Emphasis on Strategies Activating the Wnt/β‐Catenin Pathway,” Advances in Wound Care 11 (2022): 70–86. 33573472 10.1089/wound.2020.1284 PMC9831250

[14] M. Zubair and J. Ahmad , “Role of Growth Factors and Cytokines in Diabetic Foot Ulcer Healing: A Detailed Review,” Reviews in Endocrine & Metabolic Disorders 20 (2019): 207–217. 30937614 10.1007/s11154-019-09492-1

[15] K. Shakhakarmi , J. E. Seo , S. Lamichhane , C. Thapa , and S. Lee , “EGF, a Veteran of Wound Healing: Highlights on Its Mode of Action, Clinical Applications With Focus on Wound Treatment, and Recent Drug Delivery Strategies,” Archives of Pharmacal Research 46 (2023): 299–322. 36928481 10.1007/s12272-023-01444-3

[16] B. C. Oliveira , B. de Oliveira , G. Deutsch , F. S. Pessanha , and S. R. de Castilho , “Effectiveness of a Synthetic Human Recombinant Epidermal Growth Factor in Diabetic Patients Wound Healing: Pilot, Double‐Blind, Randomized Clinical Controlled Trial,” Wound Repair and Regeneration: Official Publication of the Wound Healing Society [and] the European Tissue Repair Society 29 (2021): 920–926. 10.1111/wrr.12969 34563097

[17] Y. Wei , J. Li , Y. Huang , et al., “The Clinical Effectiveness and Safety of Using Epidermal Growth Factor, Fibroblast Growth Factor and Granulocyte‐Macrophage Colony Stimulating Factor as Therapeutics in Acute Skin Wound Healing: A Systematic Review and Meta‐Analysis,” Burns & Trauma 10 (2022): tkac002. 35265723 10.1093/burnst/tkac002 PMC8900703

[18] V. Apostolopoulos , J. Bojarska , T. T. Chai , et al., “A Global Review on Short Peptides: Frontiers and Perspectives,” Molecules 26 (2021): 430. 33467522 10.3390/molecules26020430 PMC7830668

[19] S. Chang , B. D. Young , S. Li , X. Qi , J. A. Richardson , and E. N. Olson , “Histone Deacetylase 7 Maintains Vascular Integrity by Repressing Matrix Metalloproteinase 10,” Cell 126 (2006): 321–334. 16873063 10.1016/j.cell.2006.05.040

[20] J. Yang , A. Moraga , J. Xu , et al., “A Histone Deacetylase 7‐Derived Peptide Promotes Vascular Regeneration via Facilitating 14‐3‐3gamma Phosphorylation,” Stem Cells 38 (2020): 556–573. 31721359 10.1002/stem.3122 PMC7187271

[21] Y. Pan , J. Yang , Y. Wei , et al., “Histone Deacetylase 7‐Derived Peptides Play a Vital Role in Vascular Repair and Regeneration,” Advanced Science 5 (2018): 1800006. 30128229 10.1002/advs.201800006 PMC6097091

[22] X. Wang , J. Ge , E. E. Tredget , and Y. Wu , “The Mouse Excisional Wound Splinting Model, Including Applications for Stem Cell Transplantation,” Nature Protocols 8 (2013): 302–309. 23329003 10.1038/nprot.2013.002

[23] A. Vallee , Y. Lecarpentier , and J. N. Vallee , “The Key Role of the WNT/Beta‐Catenin Pathway in Metabolic Reprogramming in Cancers Under Normoxic Conditions,” Cancers 13 (2021): 5557. 34771718 10.3390/cancers13215557 PMC8582658

[24] E. Ashihara , T. Takada , and T. Maekawa , “Targeting the Canonical Wnt/Beta‐Catenin Pathway in Hematological Malignancies,” Cancer Science 106 (2015): 665–671. 25788321 10.1111/cas.12655 PMC4471797

[25] L. R. Lessey , S. C. Robinson , R. Chaudhary , and J. M. Daniel , “Adherens Junction Proteins on the Move‐From the Membrane to the Nucleus in Intestinal Diseases,” Frontiers in Cell and Developmental Biology 10 (2022): 998373. 36274850 10.3389/fcell.2022.998373 PMC9581404

[26] P. Coopman and A. Djiane , “Adherens Junction and E‐Cadherin Complex Regulation by Epithelial Polarity,” Cellular and Molecular Life Sciences 73 (2016): 3535–3553. 27151512 10.1007/s00018-016-2260-8 PMC11108514

[27] A. Hartsock and W. J. Nelson , “Adherens and Tight Junctions: Structure, Function and Connections to the Actin Cytoskeleton,” Biochimica et Biophysica Acta 1778 (2008): 660–669. 17854762 10.1016/j.bbamem.2007.07.012 PMC2682436

[28] A. Kourtidis , S. P. Ngok , and P. Z. Anastasiadis , “p120 Catenin: An Essential Regulator of Cadherin Stability, Adhesion‐Induced Signaling, and Cancer Progression,” Progress in Molecular Biology and Translational Science 116 (2013): 409–432. 23481205 10.1016/B978-0-12-394311-8.00018-2 PMC4960658

[29] R. Hayat , M. Manzoor , and A. Hussain , “Wnt Signaling Pathway: A Comprehensive Review,” Cell Biology International 46 (2022): 863–877. 35297539 10.1002/cbin.11797

[30] X. Ding , X. Wang , S. Lu , X. Gao , and S. Ju , “P120‐Catenin and Its Phosphorylation on Tyr228 Inhibits Proliferation and Invasion in Colon Adenocarcinoma Cells,” Oncotargets and Therapy 12 (2019): 10213–10225. 32063714 10.2147/OTT.S211973 PMC6884968

[31] J. Y. Hong , I. H. Oh , and P. D. McCrea , “Phosphorylation and Isoform Use in p120‐Catenin During Development and Tumorigenesis,” Biochimica et Biophysica Acta 1863 (2016): 102–114. 26477567 10.1016/j.bbamcr.2015.10.008 PMC4658284

[32] M. V. Brown , P. E. Burnett , M. F. Denning , and A. B. Reynolds , “PDGF Receptor Activation Induces P120‐Catenin Phosphorylation at Serine 879 via A PKCalpha‐Dependent Pathway,” Experimental Cell Research 315 (2009): 39–49. 18950621 10.1016/j.yexcr.2008.09.025 PMC2925109

[33] G. Chen , N. An , Y. Zhu , et al., “bFGF‐Mediated Phosphorylation of Delta‐Catenin Increases Its Protein Stability and the Ability to Induce the Nuclear Redistribution of Beta‐Catenin,” American Journal of Cancer Research 11 (2021): 3877–3892. 34522455 PMC8414378

[34] D. J. Mariner , M. A. Davis , and A. B. Reynolds , “EGFR Signaling to P120‐Catenin Through Phosphorylation at Y228,” Journal of Cell Science 117 (2004): 1339–1350. 14996911 10.1242/jcs.01001

[35] F. J. Giles , A. T. Stopeck , L. R. Silverman , et al., “SU5416, A Small Molecule Tyrosine Kinase Receptor Inhibitor, Has Biologic Activity in Patients With Refractory Acute Myeloid Leukemia or Myelodysplastic Syndromes,” Blood 102 (2003): 795–801. 12649163 10.1182/blood-2002-10-3023

[36] N. Al‐Shanti and C. E. Stewart , “Pd98059 Enhances C2 Myoblast Differentiation Through P38 Mapk Activation: A Novel Role for Pd98059,” Journal of Endocrinology 198 (2008): 243–252. 18467380 10.1677/JOE-08-0151

[37] A. Kourtidis , R. Lu , L. J. Pence , and P. Z. Anastasiadis , “A Central Role for Cadherin Signaling in Cancer,” Experimental Cell Research 358 (2017): 78–85. 28412244 10.1016/j.yexcr.2017.04.006 PMC5544584

[38] P. D. McCrea and J. I. Park , “Developmental Functions of the P120‐Catenin Sub‐Family,” Biochimica et Biophysica Acta 1773 (2007): 17–33. 16942809 10.1016/j.bbamcr.2006.06.009

[39] J. Ma and C. Wu , “Bioactive Inorganic Particles‐Based Biomaterials for Skin Tissue Engineering,” Exploration 2 (2022): 20210083. 37325498 10.1002/EXP.20210083 PMC10190985

[40] A. Joorabloo and T. Liu , “Recent Advances in Reactive Oxygen Species Scavenging Nanomaterials for Wound Healing,” Exploration 4 (2024): 20230066. 38939866 10.1002/EXP.20230066 PMC11189585

[41] H. W. An , M. Mamuti , X. Wang , et al., “Rationally Designed Modular Drug Delivery Platform Based on Intracellular Peptide Self‐Assembly,” Exploration 1 (2021): 20210153. 37323217 10.1002/EXP.20210153 PMC10190849

[42] A. P. Veith , K. Henderson , A. Spencer , A. D. Sligar , and A. B. Baker , “Therapeutic Strategies for Enhancing Angiogenesis in Wound Healing,” Advanced Drug Delivery Reviews 146 (2019): 97–125. 30267742 10.1016/j.addr.2018.09.010 PMC6435442

[43] C. Han , M. Barakat , and L. A. DiPietro , “Angiogenesis in Wound Repair: Too Much of a Good Thing?,” Cold Spring Harbor Perspectives in Biology 14 (2022): a041225. 35667793 10.1101/cshperspect.a041225 PMC9524283

[44] S. Korntner , C. Lehner , R. Gehwolf , et al., “Limiting Angiogenesis to Modulate Scar Formation,” Advanced Drug Delivery Reviews 146 (2019): 170–189. 29501628 10.1016/j.addr.2018.02.010

[45] M. Duñach , B. Del Valle‐Pérez , and A. García de Herreros , “p120‐Catenin in Canonical Wnt Signaling,” Critical Reviews in Biochemistry and Molecular Biology 52 (2017): 327–339. 28276699 10.1080/10409238.2017.1295920

[46] Z. Xie , Y. Tang , M. Q. Man , C. Shrestha , and D. D. Bikle , “p120‐Catenin is Required for Regulating Epidermal Proliferation, Differentiation, and arrier function,” Journal of Cellular Physiology 234 (2018): 427–432. 29923340 10.1002/jcp.26535 PMC9000995

[47] S. G. Dann , J. Golas , M. Miranda , et al., “p120 Catenin is a Key Effector of a Ras‐PKCvarepsilon Oncogenic Signaling Axis,” Oncogene 33 (2014): 1385–1394. 23542175 10.1038/onc.2013.91

[48] J. Yang , A. G. Bassuk , J. Merl‐Pham , et al., “Catenin Delta‐1 (CTNND1) Phosphorylation Controls the Mesenchymal to Epithelial Transition in Astrocytic Tumors,” Human Molecular Genetics 25 (2016): 4201–4210. 27516388 10.1093/hmg/ddw253 PMC5291196

