# Supplementary material for: Histone Deacetylase 7‐Derived 7‐Amino Acid Peptide Increases Skin Wound Healing via Regulating Epidermal Fibroblast Proliferation and Migration
Source: J Cell Mol Med. 2024 Nov 27;28(22):e70209. doi: 10.1111/jcmm.70209 (PMC11600263; doi:10.1111/jcmm.70209)
Supplement: Supplementary file 1 — FIGURE S1. An illustration of the procedure of the rat wound healing model. Full‐thickness excision wounds (diameter = 1.5 cm) were created on the back of SD rat. Each group was topically applied with 150 μL 25% Pluronic F‐127 gel containing either PBS, 1 μg/mL VEGF, or 50 ng/mL 7Ap peptide. n = 9. FIGURE S2. 7Ap increased HUVEC proliferation and tube formation. (A) HUVECs (5 × 10^4^/well), HEK293 (5 × 10^4^/well), and HeLa (2 × 10^4^/well) were seeded in 6‐well plates at number indicate (initial) in complete growth factor and 10 ng/mL 7Sp or 7Ap for 72 h, followed by cell number counting (final). CPD (cell population doubling) was defined as (Log10F − Log10I)/Log102, F and I indicated the final and initial number, respectively. n = 6. (B) HUVECs (1.5 × 10^4^cells/100 μL/well) in M199 medium supplemented with 10% FBS were seeded in 96 well plates containing 80 μL/well growth factor reduced Matrigel with 10 ng/mL 7Sp or 7Ap or 50 ng/mL VEGF, followed by imaging at 5 and 24 h. Left: representative images. Right: branch numbers analysis from views indicated. ns: no significant. ***:p < 0.001. [file JCMM-28-e70209-s001.docx]

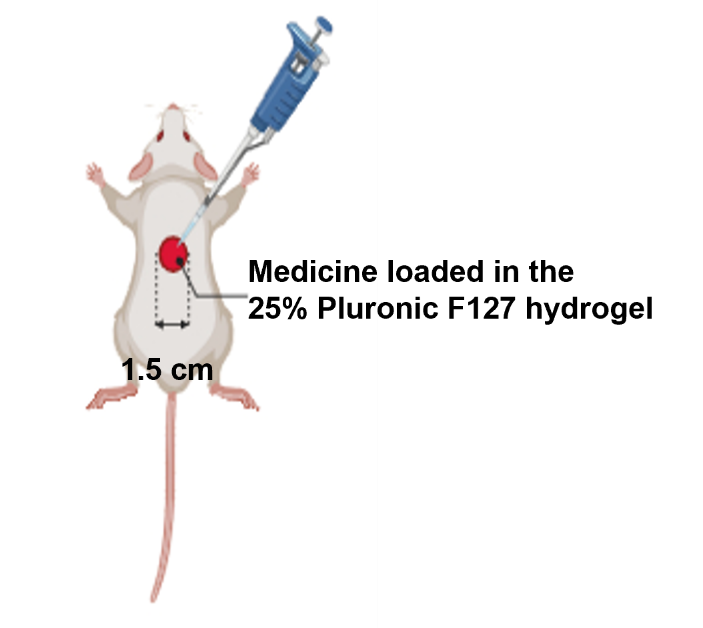


**FIGURE S1 An illustration of the procedure of the rat wound healing model**. Full-thickness excision wounds (diameter = 1.5 cm) were created on the back of SD rat. Each group was topically applied with 150 μl 25% Pluronic® F-127 gel containing either PBS, 1 μg/ml VEGF, or 50 ng/ml 7Ap peptide. n=9.

**
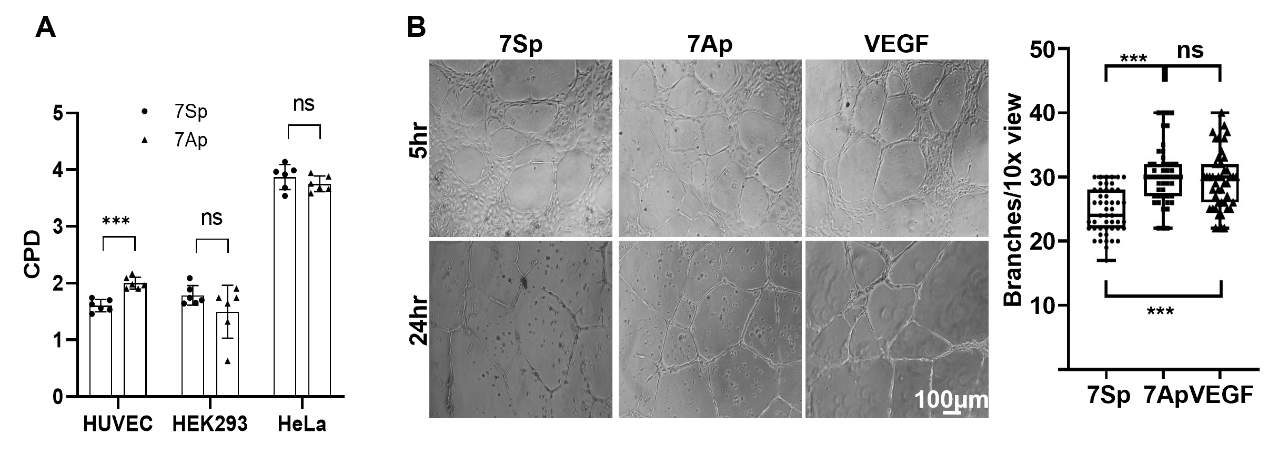
**

**FIGURE S2 7Ap increased HUVEC proliferation and tube formation.** (**A**) HUVECs (5x10^4^/well), HEK293 (5x10^4^/well), and HeLa (2x10^4^/well) were seeded in 6-well plates at number indicate (initial) in complete growth factor and 10ng/ml 7Sp or 7Ap for 72hr, followed by cell number counting (final). CPD (cell population doubling) was defined as (Log_10_F-Log_10_I)/Log_10_2, F and I indicated the final and initial number, respectively. n=6. (**B**) HUVECs (1.5x10^4^cells/100μl/well) in M199 medium supplemented with 10% FBS were seeded in 96 well plates containing 80μl/well growth factor reduced Matrigel with 10ng/ml 7Sp or 7Ap or 50ng/ml VEGF, followed by imaging at 5hr and 24hr. Left: representative images. Right: branch numbers analysis from views indicated. ns: no significant. ***：*p*<0.001.
